# The Story of the Insane from Year to Year

**Published:** 1892-06-18

**Authors:** 


### The Story of the Insane from Year to Year.

(Fourth Series.)

### CAPE OF GOOD HOPE ASYLUMS.

On the first of January, 1890, there were in the asylums and lunatic hospitals of Cape Colony, 572 insane persons, and by the end of the year the number had risen to 595. Of these 310 were of European birth or descent and 285 were "coloured"; that is, they were, with a few exceptions, natives of Cape Colony or of other parts of South Africa. The lunatics are confined in five separate institutions. The Robben Island Asylum contains 225; the Grahams Town Asylum, 171; the Old Somerset Hospital, 110; the Port Alfred Asylum, 82, and the Sick Hospital, 7. Dr. Dodds, the Inspector of Asylums for the Colony, groups the asylums, so to speak, under one head, and presents us with an extremely clear and well-arranged report. He sees the short-comings of the institutions he has to inspect; evidently does his best to remedy defects, and we notice that considerable advance has lately been made in many directions. One of the most pressing things is the lack of asylum accommodation; so much is this the case that 117 lunatics were treated in the prisons of the Colony, a fact which our English readers will find a difficulty in realizing. It is true that the majority of these were transferred to the asylums; but the year closed with 29 insane persons domiciled in the gaols of Cape Colony. An appendix contains special reports on the individual institutions. We extract the following from the Grahams Town Asylum Report: "It is found that among the Europeans the recovery rate for the men was 29·17 per cent., and for the women only 28·17; whereas amongst natives the recovery rates were respectively 68·75 and 51·14. If statistics prove anything, these figures show that the native insane get well sooner, and the proportion of recoveries among them is greater than among the Europeans, and this is, undoubtedly, due to the simpler forms of insanity found in the natives compared with the more serious forms which it assumes when it occurs in persons of higher mental development."

### CENTRAL INDIANA HOSPITAL FOR THE INSANE.

England is unfortunately no exception as regards the enormous size of many of her asylums. In the Indiana Hospital not fewer than 2,100 lunatics were under treatment during the year. At the beginning of the fiscal year 1,526 were in residence, and at the close this number had risen to 1,557. The admissions were 574; the recoveries, 242; and the deaths, 85. Hereditary tendency was the assigned cause of the insanity in 39 cases, and drink sent 19 to the asylum. These, together, only account for 58 of the admissions, and this must fall far short of the truth. Indeed, we notice that 204 are put down as "unknown," so that the table given on page 22 is practically useless. Consumption was the cause of death in 17 instances. Dr. Wright tells us that he has "made only such use of restraint and seclusion as seemed absolutely necessary." But what does he consider absolutely necessary? We should much like to know what our cousins think on these subjects. The asylum is well officered, there being five assistant physicians.

### STATE LUNATIC HOSPITAL AT HARRISBURG, PENNSYLVANIA.

This is the forty-first annual report. In the first complete year of its existence it had only 106 patients, and now it has 803, and a rate of increase as great as any of our English asylums. The admissions were 228, the recoveries 64, and the deaths 82. Dr. Gerhard tells us that during the last decade a larger number of incurable patients have been sent to the State Hospital than formerly. Chronic cases from the almshouses, including idiots and imbeciles, and further that there has been a great increase in the number of paralytics. As a natural result, the percentage of recoveries has decreased, and the percentage of deaths has increased. There is evidently a strong family likeness in asylums, for the above sentences would apply to one-half of our English county asylums. We notice that influenza is the assigned cause of the insanity in six of the year's admissions; but, even when allowance is made for the number of patients in whom the cause of insanity was unknown, neither drink nor hereditary influence seems to play so important a part as in this country. There are four assistant physicians, two being ladies.

### NEW ZEALAND ASYLUMS.

In the various asylums of this colony, the numbers rose during the year from 1,761 to 1,797, being an increase of only 36. The patients were distributed as follows: In the Auckland Asylum, 389; in Christchurch, 368; in Seacliffe, 496; in Hokitaka, 105; in Nelson, 98; in Wellington, 272; and in the Ashburn Private Asylum, 33. The proportion of the insane to the population generally is in New Zealand one in 351, and in England one in 341. The admissions were 390; the percentage of recoveries calculated in the usual English manner, was 47·69, and the percentage of deaths was 6·29. Drink heads the list of causes with 53, and hereditary influence comes second, accounting for 36 of the year's admissions. Consumption is responsible for only six deaths out of a total of 111. The Inspector of New Zealand asylums is Dr. Macgregor, and his reports are very interesting in many ways. The average cost per head per annum throughout the asylums is £18 18s. 3d.

